# Predicting Agitation-Sedation Levels in Intensive Care Unit Patients: Development of an Ensemble Model

**DOI:** 10.2196/63601

**Published:** 2025-02-26

**Authors:** Pei-Yu Dai, Pei-Yi Lin, Ruey-Kai Sheu, Shu-Fang Liu, Yu-Cheng Wu, Chieh-Liang Wu, Wei-Lin Chen, Chien-Chung Huang, Guan-Yin Lin, Lun-Chi Chen

**Affiliations:** 1 Department of Digital Medicine, Taichung Veterans General Hospital, Taichung, Taiwan; 2 Department of Nursing, Taichung Veterans General Hospital, 1650 Taiwan Boulevard Sect. 4, Taichung, 40705, Taiwan; 3 Department of Computer Science, Tunghai University, Taichung, Taiwan; 4 Department of Critical Care Medicine, Taichung Veterans General Hospital, No 1650, Section 4, Taiwan Boulevard, Xitan District, Taichung City, 407219, Taiwan, 886-04-23592525 #2002; 5 Department of Post-Baccalaureate Medicine, College of Medicine, National Chung Hsing University, Taichung, Taiwan; 6 Computer & Communications Center, Taichung Veterans General Hospital, Taichung, Taiwan; 7 College of Engineering, Tunghai University, Taichung, Taiwan

**Keywords:** intensive care units, ICU, agitation, sedation, ensemble learning, machine learning, ML, artificial intelligence, AI, patient safety, efficiency, automation, ICU care, ensemble model, learning model, explanatory analysis

## Abstract

**Background:**

Agitation and sedation management is critical in intensive care as it affects patient safety. Traditional nursing assessments suffer from low frequency and subjectivity. Automating these assessments can boost intensive care unit (ICU) efficiency, treatment capacity, and patient safety.

**Objectives:**

The aim of this study was to develop a machine-learning based assessment of agitation and sedation.

**Methods:**

Using data from the Taichung Veterans General Hospital ICU database (2020), an ensemble learning model was developed for classifying the levels of agitation and sedation. Different ensemble learning model sequences were compared. In addition, an interpretable artificial intelligence approach, SHAP (Shapley additive explanations), was employed for explanatory analysis.

**Results:**

With 20 features and 121,303 data points, the random forest model achieved high area under the curve values across all models (sedation classification: 0.97; agitation classification: 0.88). The ensemble learning model enhanced agitation sensitivity (0.82) while maintaining high AUC values across all categories (all >0.82). The model explanations aligned with clinical experience.

**Conclusions:**

This study proposes an ICU agitation-sedation assessment automation using machine learning, enhancing efficiency and safety. Ensemble learning improves agitation sensitivity while maintaining accuracy. Real-time monitoring and future digital integration have the potential for advancements in intensive care.

## Introduction

Patients admitted to intensive care units (ICUs) often experience various clinical problems, such as pain, agitation, and delirium. Agitation refers to physical restlessness due to treatment discomfort or delirium; this condition cannot be self-controlled [1]. Agitation is common in patients in ICUs; most of these patients (71%) exhibit agitation on approximately 58% of their total inpatient days [2-4]. Agitation can lead to the accidental removal of tubes and catheters, compromising patient safety, extending ICU stays, and causing complications [5]. Throughout the treatment period, nurses must regularly evaluate the levels of agitation and sedation and titrate the dosages of sedatives accordingly for patient care.

Various scales have been developed for measuring sedation effects. Among them, the Richmond Agitation-Sedation Scale (RASS) is the most reliable and effective [6]. This scale was developed by a multidisciplinary team at Virginia Commonwealth University in Richmond. It employs a simple and clearly defined scoring system with distinct standards for measuring the levels of sedation and agitation. Agitation and sedation levels are represented by positive and negative scores, respectively. The RASS assessment is performed by nurses every few hours, which consumes their significant work time. Reducing the time required for RASS evaluations could increase ICU treatment capacity, thereby improving care quality and patient safety.

However, this scale has some disadvantages, such as low evaluation frequency and high subjectivity, due to variations in patient evaluation standards among medical personnel. Occasionally, nurses may have insufficient knowledge about delirium, which can increase the risk of incorrect evaluations by 20 times [7]. Furthermore, errors in evaluation of patient conditions may result in excessive or insufficient sedation. These issues can be attributed to the subjectivity and uncertainties in the RASS evaluation process, which relies on patients’ audiovisual responses, making it is unsuitable for those with severe audiovisual impairment [8]. The RASS facilitates intermittent measurement of agitation levels and assessment of patient behavior; however, unlike activity monitors, it cannot assist in the continuous monitoring of agitation levels [9].

The study’s aim was to develop an ensemble learning model for the continuous evaluation of agitation and sedation levels in patients admitted to ICUs. The model is expected to facilitate patient monitoring, provide early warnings about patient behavior, increase assessment frequency, and enable automatic evaluation of patient conditions with treatment suggestions. We believe that this novel design could improve the clinical monitoring of agitation and sedation levels in patients in ICUs, enhance the quality of medical care, and reduce the wastage of medical resources.

## Methods

### Setting

Taichung Veterans General Hospital (TCVGH) is a 1530-bed medical center in central Taiwan with 7 ICUs comprising a total of 138 beds. We obtained access to the critical care database (AI-111010) of the AI Center of TCVGH. The following data were collected: basic information, disease severity, ventilator use, blood biochemistry, vital signs, catheter types, and medication records.

### Research Framework

**Figure 1.**
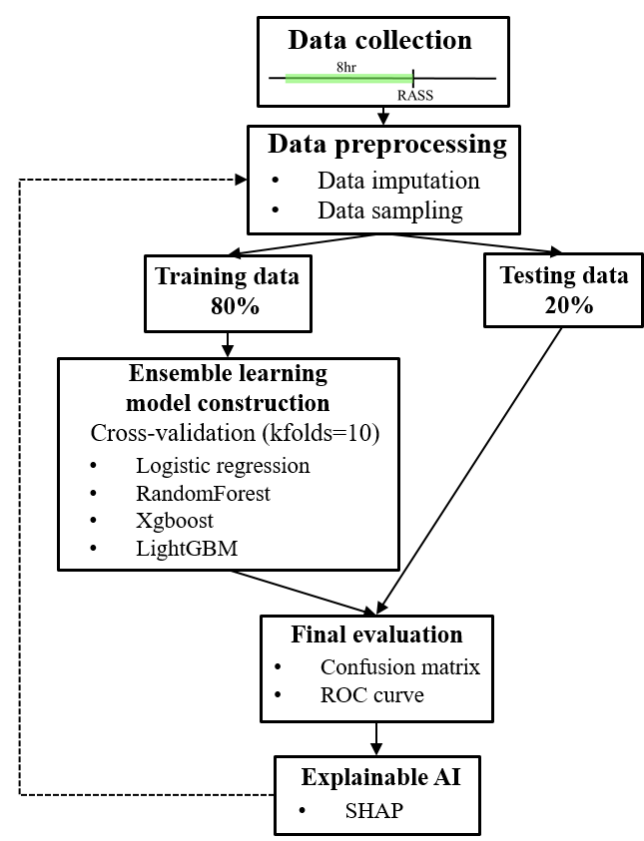
Research framework consisted of 5 steps: (1) data collection in the ICU, (2) data preprocessing (data imputation, data sampling), (3) ensemble learning model construction, (4) final evaluation, (5) explainable AI. AI: artificial intelligence; RASS: Richmond Agitation–Sedation scale; ROC: receiver operating characteristic; SHAP: Shapley additive explanations.

The study consisted of five major steps: (1) data collection in the ICU, (2) data preprocessing (data imputation and data sampling), (3) ensemble learning model construction, (4) final evaluation, and (5) implementation of explainable AI (Figure 1).

### Data Preprocessing

#### Data Imputation

Basic information includes patients’ age. Predicting RASS in older patients is more challenging. Regarding disease severity, patients’ acute physiology and chronic health evaluation (APACHE II) scores may impact their predicted RASS scores [6,10]. Missing data were imputed using average values.

In addition, the use of ventilators was considered. Ventilator modes were categorized into 3 conditions: no ventilator use, noninvasive ventilator use, and invasive ventilator use. Invasive ventilators may cause discomfort, indirectly affecting RASS scores [8,10-12]. Furthermore, PAW (average airway pressure) values, a ventilator parameter, were estimated accordingly. For patients on ventilators, the average value from all ventilator-wearing patients was used to impute missing data, whereas patients without ventilators were assigned normal random values.

In the category of blood biochemistry, features such as creatinine, lactate, and glucose were included. These were directly associated with patients’ physiological conditions [10]. Typically, blood tests were conducted weekly within a 7-day data window. When no blood test data were available within this timeframe, indicating stable patient condition, normal random values were used for imputation.

Vital signs, including blood pressure, pulse rate, and respiratory rate, were indirectly associated with changes in patients’ RASS scores [13]. Since vital signs were densely and continuously monitored features, adjacent values were used to fill in missing data directly.

Medication records included the type and dosage of sedatives and analgesics administered to patients, such as benzodiazepine sedatives, muscle relaxants, opioid-related analgesics, antipsychotics, hypnotics, and anesthetics. These drugs can directly or indirectly influence patients’ consciousness levels and RASS score changes. Due to the varying recording methods for drug dosage across different types of medications, establishing a consistent standard was challenging. Therefore, in this study, the presence or absence of records indicating the use of muscle relaxants or sedative-hypnotics within the past 8 hours was used as a feature for RASS assessment.

#### Data Sampling

To address severe data imbalance issues (oversedation: 43,199 cases, maintain range: 77,290 cases, agitation: 814 cases), this study employed data oversampling and undersampling techniques to enhance model learning effectiveness. In addition, discussions with clinical experts were conducted to determine the most suitable sampling approach.

### Model Construction

Based on clinical care experience, this study proposed an ensemble learning model that integrates two submodels—sedation and agitation—to classify events into oversedation (RASS -5 to -2), maintain range (clinically expected maintenance RASS -1 to 1), and agitation (RASS 2 to 4).

**Figure 2.**
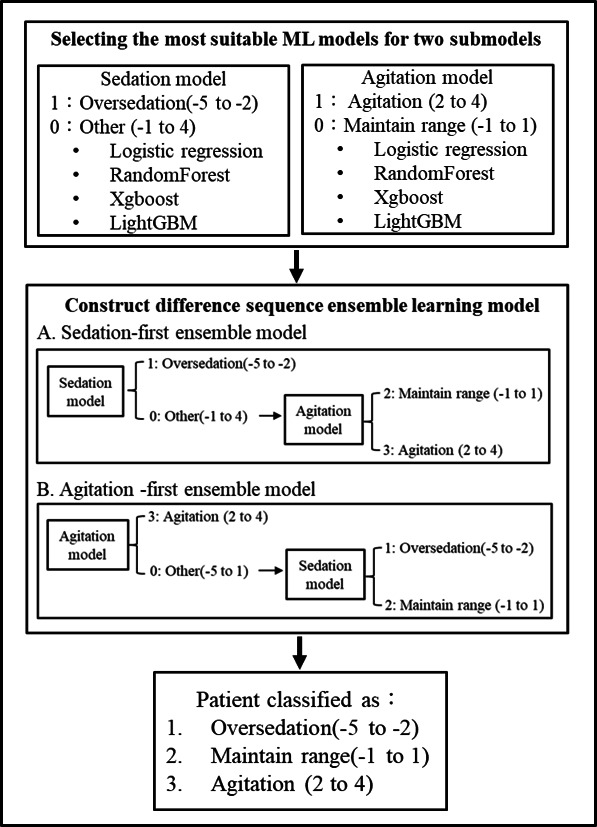
Ensemble model construction consisted of 3 steps: (1) Choose a more suitable ML model, (2) Construct difference sequence ensemble learning model, (3) Classify patient into three categories. ML: machine learning

First, the sedation and agitation models were constructed using 4 ML algorithms: logistic regression, random forest, XGBoost, and LightGBM. The algorithm with the best performance was selected as the foundation for both the sedation and agitation models. Subsequently, the two submodels were combined into 2 sequential ensemble learning models: the sedation-first ensemble model and the agitation-first ensemble model. In the sedation-first model, the sedation model was first used to distinguish “oversedation” from “other,” and the remaining categories were then input into the agitation model to further differentiate between “maintain range” and “agitation.” Conversely, in the agitation-first model, the agitation model was first applied to separate “agitation” from “other,” and the remaining categories were then passed into the sedation model to classify “oversedation” and “maintain range.” (Figure 2).

### Final Evaluation

We used confusion matrices and the receiver operating characteristic (ROC) curves as indicators to evaluate model accuracy, precision, recall, F1-score, and area under the curve. The ROC curves helped to compare sensitivity with specificity. Effective models exhibited high sensitivity and specificity, resulting in high area under the curve values.

### Explainable AI

Explainable Artificial Intelligence (XAI) was applied, making the ML system transparent. The top 20 features were selected, and Shapley additive explanations and partial dependence plots were used to visualize their contributions, aiding in understanding the model’s decision-making process. Clinical personnel could use this information to offer patient-specific evaluations or decision-making suggestions.

### Statistics

This study used statistical methods, including mean (SD) and *t*-test, for numerical data analysis and observation. These methods were used to describe dataset central tendencies, assess variability, and compare group differences. Proportions of each category were also calculated for categorical data. They helped to extract meaningful information and interpret research results.

### Ethical Considerations

This study was approved by the TCVGH Institutional Review Board (CE22484A). Informed consent was obtained, with participants given the option to withdraw at any time. For secondary analyses, original consent covered data reuse without additional approval. Data were anonymized to protect privacy, and strict security measures were applied.

## Results

### Data Collection

**Figure 3.**
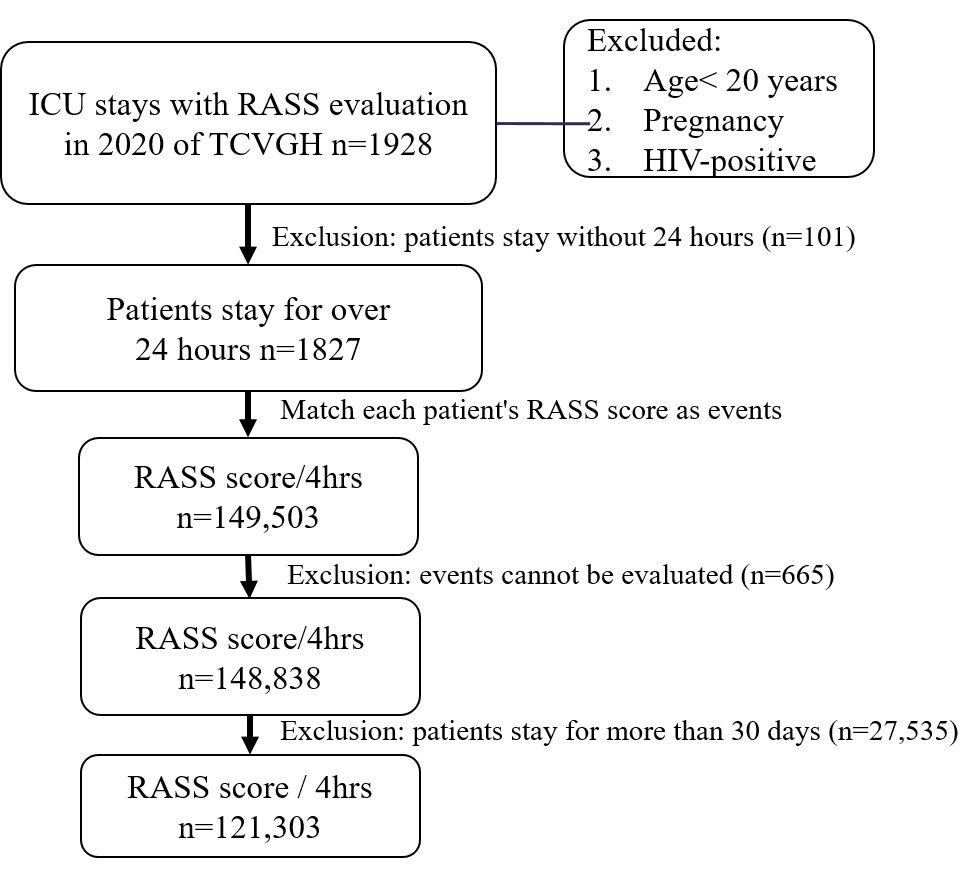
Flowchart of subject enrollment. ICU: intensive care unit; RASS: Richmond Agitation–sedation scale; TCVGH: Taichung Veterans General Hospital.

This study collected data from adult patients (aged≥20 years) admitted to the ICU at TCVGH between January 1 and December 31, 2020, with an ICU stay lasting more than 24 hours. Every 4-hour RASS assessment (with increased frequency depending on the patient’s condition) was considered as a classification event, with events marked as not assessable excluded due to concurrent procedures. Since the average ICU stay did not exceed 30 days, events from ICU stays longer than 30 days were also excluded. A total of 121,306 events were collected, with an average of 108 events per patient (range: 6 to 186). The machine learning (ML) model was trained using data from the 8 hours prior to each event, including ventilator parameters, vital signs, and medication records, along with laboratory biochemical data from the previous week (Figure 3).

#### Feature Selection

Based on the literature and clinical experience, the feature selection process in this study involved in-depth discussions with physicians and nurses. During this process, the missing rate of each feature was considered, and features with a missing rate exceeding 20% were excluded. Finally, 23 features were selected, and appropriate imputation methods were applied (Table 1). Categorical features (eg, ventilator modes and medications) were defined based on the presence of recorded events within the past 8 hours. Continuous features such as vital signs and ventilator parameters were calculated as the average values over the past 8 hours, while laboratory test features were based on the most recent record within the past week.

**Table 1.** All features used in model training, including their respective units, missing rates, and data imputation methods aligned with clinical requirements.

Projects and features	Unit	Missing rate (%)	Imputation method
Basic Information			
Age	years	0.0	None
Disease severity			
APACHE^a^ II	score	2.4	Average
Use of ventilator	—^b^	0.0	None
Ventilation Mode	—		
No ventilation (0)	—		
Noninvasive ventilation (1)	—		
Invasive ventilation (2)	—		
Average airway pressure (Paw)	cmH_2_O	5.0	Average/Normal Range (Random)
Blood biochemical test data			Normal Range (Random)
BUN^c^	mg/dL	4.8	
Creatinine	mg/dL	2.0	
Glucose (One touch)	mg/dL	2.7	
Bicarbonate (HCO_3_)	mmol/L	11.8	
Hematocrit (Hct)	%	12.7	
Hemoglobin (Hgb)	g/dL	0.2	
Potassium (K)	mEq/L	0.2	
Lactate	mg/dl	17.3	
Phytohemagglutinin (PH_A)	value	12.7	
Sodium (Na)	mEq/L	12.7	
Platelets (PLT)	thou/mm³	0.2	
Partial pressure of oxygen (PO_2_)	mmHg	4.1	
White blood cells (WBC)	thou/mm³	0.3	
Vital signs			Pre and post values
Systolic blood pressure (SBP)	mmHg	0.1	
Diastolic blood pressure (DBP)	mmHg	0.1	
Respiratory rate (RR)	bpm	0.1	
Blood oxygen saturation (SPO_2_)	%	0.0	
Pulse	bpm	0.0	
Medication records		0.0	None
Medicine	—		
No (0)			
Yes (1)			

a APACHE: acute physiology and chronic health evaluation.

b Not applicable.

c BUN: blood urea nitrogen.

### Description of the Study Population

Statistical analysis indicated that nearly all features significantly affect the level of agitation-sedation. Patients with oversedation typically exhibited higher disease severity, lower blood oxygen levels, and a higher proportion of invasive ventilator use. In contrast, patients with agitation showed higher vital sign values and a greater proportion of sedative medication usage (Table 2).

**Table 2.** Statistical analysis of datasets used to train the two models. Proportion: Percentage of Population

	Overall (N=121,303)	Oversedation(n=43,199)	Maintain range(n=77,290)	Agitation (n=814)	*P* value
Numerical features, mean (SD)					
Age	67.5 (14.9)	68.21 (5.2)	67.2 (14.8)	66.1 (15.3)	<.001
APACHE^a^ II	23.9 (7)	26.4 (6.6)	22.5 (6.8)	23.2 (6.2)	<.001
Average airway pressure (Paw)	22.0 (5.7)	23.6 (6.2)	21.1 (5.1)	21.3 (4.9)	<.001
BUN^b^	39.8 (30.6)	45.1 (33.7)	36.8 (28.4)	34.7 (26.7)	<.001
Creatinine	2.0 (2.2)	2.1 (2.2)	1.9 (2.2)	1.9 (2.2)	<.001
Glucose (One touch)	168.8 (49.4)	175.5 (53.1)	165.2 (46.8)	162.9 (46.9)	<.001
Bicarbonate (HCO_3_)	24.1 (4.7)	23.4 (5.1)	24.5 (4.4)	24.8 (4.7)	<.001
Hematocrit (Hct)	30.9 (7.1)	30.9 (7.6)	30.9 (6.8)	32.2 (7)	.14
Hemoglobin (Hgb)	9.7 (1.9)	9.6 (2)	9.8 (1.8)	10.1 (1.9)	<.001
Potassium (K)	3.9 (0.6)	4.0 (0.6)	3.9 (0.5)	3.9 (0.6)	<.001
Lactate	17.7 (16.6)	22.4 (21.4)	15.1 (12.4)	18.0 (20.4)	<.001
Phytohemagglutinin (PH_A)	7.4 (0.1)	7.4 (0.1)	7.4 (0.1)	7.4 (0.1)	<.001
Sodium (NA)	140.3 (6.5)	141.0 (6.9)	139.9 (6.2)	141.4 (6.4)	<.001
Platelets (PLT)	187.8 (114.5)	175.3 (108.2)	194.7 (117.3)	201.1 (115.5)	<.001
Partial pressure of oxygen (PO_2_)	124.2 (50.2)	120.4 (49.6)	126.3 (50.4)	121.5 (53.7)	<.001
White blood cells (WBC)	12394.1 (12277)	13764.4 (15679.1)	11636.5 (9854)	11601.5 (6364.6)	<.001
Systolic blood pressure (SBP)	123.2 (19.4)	119.5 (19.7)	125.2 (18.9)	126.2 (18.5)	<.001
Diastolic blood pressure (DBP)	70.1 (12.7)	67.4 (13)	71.6 (12.2)	73.7 (11.8)	<.001
Respiratory rate (RR)	18.8 (4.2)	19.7 (4.9)	18.2 (3.7)	19.1 (3.9)	<.001
SPO_2_	97.2 (7.2)	95.9 (10.9)	97.9 (3.7)	97.2 4)	<.001
Pulse	88.7 (17.8)	90.4 (19.3)	87.7 (16.8)	93.2 (18.5)	<.001
Categorical features, n (%)			
Medicine (Yes)	26,787 (22%)	8,601 (20%)	17,658 (23%)	528 (65%)	<.001
Invasive ventilation	92,257 (76%)	40,168 (93%)	51,505 (67%)	584 (72%)	<.001
Noninvasive ventilation	5937 (5%)	587 (1%)	5295 (7%)	55 (7%)	<.001
No ventilation	23,109 (19%)	2444 (6%)	20,490 (26%)	175 (21%)	<.001

a APACHE: acute physiology and chronic health evaluation.

b BUN: blood urea nitrogen.

### Model Development and Validation

#### Data Sampling of Agitation Classification

Given the significant class imbalance observed (Table 2), we explored various data sampling methods to enhance the model’s sensitivity (recall) for detecting agitated patients. Among these methods, the undersampling approach demonstrated the most notable performance, achieving a sensitivity of 0.82 (Table 3). Consequently, we selected the undersampling method as the data processing strategy.

**Table 3.** Random forest model performance of different sampling methods for agitation category patients.

Classification	Sampling method (number of patients)	Precision	Recall	F1 score
Agitation Model	Non (n=77288, 814)	0.81	0.26	0.39
Undersampling (n=642,642)	0.03	0.82	0.06
SMOTE^a^ (n=61834, 61834)	0.28	0.24	0.26

a SMOTE: synthetic minority oversampling technique

#### Performance Comparison of Two Submodels in ML Models

The results of the confusion matrices and ROC curves for the 4 ML models (as shown in Table 4) indicate that the random forest model outperformed the others in both the sedation prediction (accuracy: 0.92, AUC: 0.96) and agitation prediction (accuracy: 0.80, AUC: 0.88). Additionally, the random forest model exhibited superior sensitivity (recall) for detecting agitated patients. Consequently, we selected the random forest model as the foundation for constructing various ensemble learning frameworks to facilitate further analysis and applications.

**Table 4.** Four different ML models performance comparison of sedation classification and agitation classification.

Classification and models	Accuracy	Precision	Recall	AUC^a^	Cross-validation Avg-ACC^b^ (kfold=10)
Sedation					
Logistic regression	0.72	0.71	0.65	0.71	0.71
Random forest	0.92	0.91	0.91	0.96	0.92
XGBoost^c^	0.92	0.91	0.91	0.94	0.90
LGBM^d^	0.83	0.81	0.83	0.90	0.84
Agitation					
Logistic regression	0.64	0.51	0.61	0.72	0.66
Random forest	0.80	0.03	0.82	0.88	0.77
XGBoost	0.76	0.51	0.76	0.84	0.73
LGBM	0.75	0.51	0.77	0.85	0.75

a AUC: area under the curve.

b ACC: accuracy.

c XGBoost: extreme gradient boosting.

d LGBM: light gradient-boosting machine.

#### Ensemble Learning Model Performance Comparison

The performance results of different sequences of ensemble learning models indicate that prioritizing the identification of agitated patients is more effective in improving sensitivity (recall: 0.82) compared to strategies that first identify oversedated patients. Furthermore, the AUC for all 3 states remained above 0.82 (as shown in Table 5. Given the higher immediate risk associated with agitated patients, the agitation-first ensemble model was selected as the most suitable approach.

**Table 5.** Comparison of performance in classifying the three categories among ensemble learning models with different sequences.

Classification sequence and categories	Accuracy	Precision	Recall	AUC^a^
Sedation-first ensemble model	0.79			
Oversedation		0.91	0.84	0.90
Maintain range		0.92	0.84	0.82
Agitation		0.03	0.76	0.81
Agitation-first ensemble model	0.75			
Oversedation		0.92	0.73	0.85
Maintain range		0.92	0.76	0.82
Agitation		0.03	0.82	0.82

a AUC: area under the curve.

### Explainable AI (XAI)

**Figure 4.**
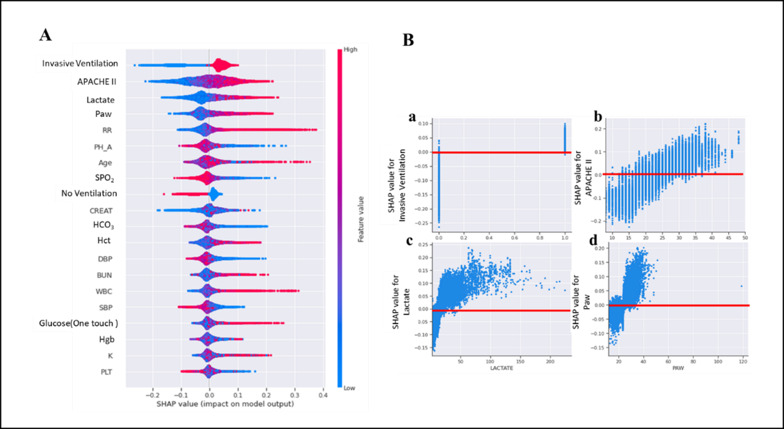
The explanations of features contribution to sedation classification. (A) The attributes of the features in the model. Each line represents a feature, and the abscissa is the SHAP value. Red dots represent higher feature values, and blue dots represent lower feature values. (B) SHAP dependence plot for the top 4 clinical features contributing to the model. APACHE: acute physiology and chronic health evaluation; Paw: average airway pressure.

The top 4 features that contribute the most to sedation classification are the use of an invasive ventilator (invasive ventilation), APACHE II score, lactate level (LACTATE), and average airway pressure (PAW). A high APACHE II score indicates a high likelihood of oversedation (Figure 4A). The dependency graph of the first 4 features represents how each feature affects the classification results. In most cases, an APACHE II score>29 was positively associated with oversedation (Figure 4B).

**Figure 5.**
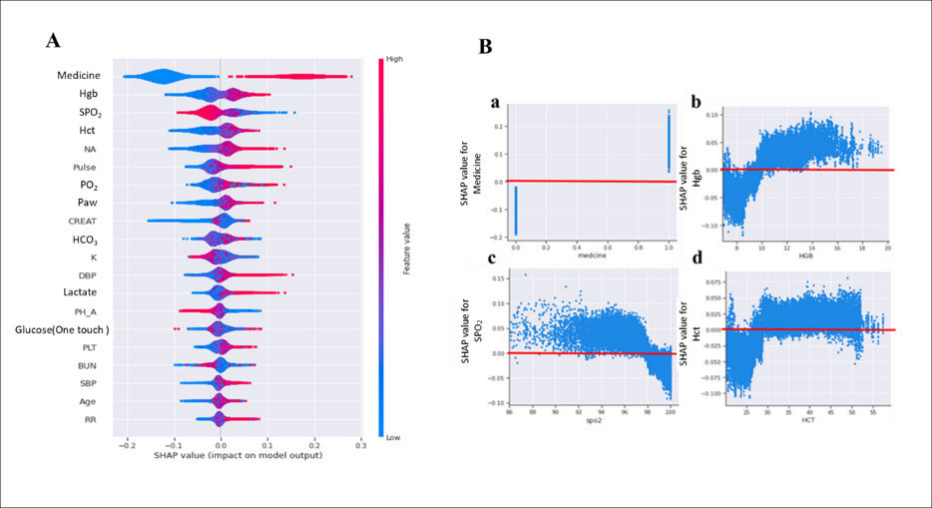
The explanations of features contribution to agitation classification. (A) The attributes of the features in the model. Each line represents a feature, and the abscissa is the SHAP value. Red dots represent higher feature values, and blue dots represent lower feature values. (B) SHAP dependence plot for the top 4 clinical features contributing to the model. HCT: hematocrit; Hgb: hemoglobin; SpO_2_: blood oxygen saturation.

Patients who used sedatives were more likely to experience agitation. Patients with a high hemoglobin level were more likely to experience agitation. The dependence plots of the top 4 features indicated that sedative use was positively correlated with agitation. Hemoglobin levels >10 g/dL and≤10 g/dL were positively correlated with agitation and maintained sedation range, respectively (Figure 5).

Overall, patients on mechanical ventilation were mostly sedated, with those in the maintain range exhibiting more stable blood test results and vital signs compared to oversedated or agitated patients.

## Discussion

### Principal findings

This study successfully developed a model for automating RASS-like agitation-sedation evaluation. Automated agitation-sedation evaluation could be an alternative to RASS and play a crucial role in enhancing ICU efficiency, ultimately improving health care outcomes, care quality, and patient safety.

Machine learning aids ICU personnel in the early detection of high-risk events [14]. Previous studies have used ML to predict mortality rates in ICU patients with acute kidney injury, predict postoperative sepsis mortality rates, and forecast extubation failure in the ICU [15-17]. However, studies on ML for agitation-sedation evaluation in ICU patients are limited.

Zhang et al [18] ensemble 4 machine-learning methods for predicting agitation in ventilated patients under light sedation for 24 hours. However, their ensemble model was limited to predicting agitation in patients with invasive ventilator support under light sedation for 24 hours. Other researchers have proposed using patient body and facial image monitoring for agitation detection [19,20]. However, image monitoring faces challenges such as data acquisition, clinical environment influences, workflow integration, and system installation. Therefore, in addition to the imaging model developed by our research team [21], we have created another model using commonly available feature data in most hospitals, serving as an alternative solution when image monitoring is not feasible.

The study employs 2 ML models for ensemble learning to automate RASS assessment. By using undersampling and adjusting the classification sequence, the sensitivity for detecting agitation is enhanced. Although models with higher sensitivity may reduce the accuracy of classifying over-sedated patients, they perform better in mitigating immediate risks. Traditional methods, which involve manual assessments every 4 hours, result in insufficient monitoring, especially for patients who may experience multiple episodes of agitation within a short period. In contrast, our health care information system can transmit data in real time, perform inference every hour, and adjust inference frequency based on clinical needs, enabling more intensive monitoring. This allows clinicians to track patient conditions and respond promptly to changes continuously.

Understanding algorithmic predictions is crucial in clinical practice. Due to the lack of explanations in the decision-making process, clinicians often distrust black-box models. Explainable artificial intelligence enhances transparency, aiding in the development of reliable decision models [22-24]. Continuous analgesics and sedatives ensure optimal gas exchange between patients and ventilators, making deep sedation important during this period [25]. For patients with stable parameters such as hemoglobin, hematocrit, blood oxygen saturation, and creatinine, the goal is maintaining light sedation (RASS score 0 to −2) [26]. It has been demonstrated that the interpretability of the model aligns with clinical experience.

This study acknowledges its limitations, particularly the imbalance in case numbers due to the high risk of agitation in patients. Especially for agitated individuals, the model’s precision of 0.03 suggests there is room for improvement. In future clinical applications, we plan to adjust the decision threshold based on specific needs to balance sensitivity and specificity and reduce false positives. Additionally, the study lacks observations of potential drug overdoses at different sedation levels. Future efforts will focus on integrating this model with digital imaging monitoring and a comprehensive drug dosage system. Real-time monitoring will help identify patient conditions, guide prescription adjustments, and accelerate recovery, ultimately supporting early intervention, ensuring patient safety, and improving the quality of intensive care.

### Conclusions

This study proposes using ML technology to achieve automated RASS-based assessments in ICU settings, enhancing clinical efficiency, and patient safety. Our integrated learning model, combined with the hospital information system, enables real-time data transmission, supports intensive monitoring, and facilitates continuous tracking of patient conditions. The system automatically categorizes patients into three groups, significantly improving sensitivity in detecting agitation categories. This innovative approach not only alleviates the workload of health care professionals but also advances the precision and intelligence of critical care management.

## Abbreviations

APACHE: acute physiology and chronic health evaluation
AUC: area under the curve
ICU: intensive care unit
ML: machine learning
RASS: Richmond Agitation-Sedation Scale
ROC: Receiver Operating Characteristic
TCVGH: Taichung Veterans General Hospital
